# Recurrent trichilemmal carcinoma of the periorbital region treated with IMRT radiotherapy: A case report and a review of literature

**DOI:** 10.1097/MD.0000000000034038

**Published:** 2023-06-16

**Authors:** Shan Gao, Qin Xu, Yanli Lan, Lang He

**Affiliations:** a Cancer Prevention and Treatment Institute of Chengdu, Department of Otolaryngology-Head and Neck Surgery, Chengdu, China; b Cancer Prevention and Treatment Institute of Chengdu, Department of Oncology, Chengdu Fifth People’s Hospital (The Second Clinical Medical College, Affiliated Fifth People’s Hospital of Chengdu University of Traditional Chinese Medicine), Chengdu, China.

**Keywords:** cutaneous adnexal tumor, radiotherapy, recurrent, the periorbital region, trichilemmal carcinoma

## Abstract

Trichilemmal carcinoma (TLC) is a rare malignant cutaneous adnexal tumor usually accept surgery. This report describes an elderly patient with recurrence TLC of the periorbital region after surgery who was subsequently treated with IMRT radiotherapy. After 2-years follow-up visit, there was no progress or metastasis.

**Introduction:**

TLC is a rare malignant cutaneous adnexal tumor. It usually occurs on sun-exposed areas in elderly people but rarely occurs in the periorbital region. Most cases accept surgery or micrographic Mohs surgery. Recurrence or metastasis of this neoplasm was seldom reported in the medical literature after enough tumor-free margin surgery. And radiotherapy was seldom reported in the treatment for patients of TLC.

**Patient concerns:**

Here we report an elderly patient with recurrence TLC of the periorbital region after surgery who was subsequently treated with radiotherapy with a total dose of 66 Gy. Two years later, the patient was admitted head, neck, chest, abdomen CT scan, and no progress or metastasis was detected after 2-years follow-up.

**Diagnosis:**

Trichilemmal carcinoma of the periorbital region.

**Interventions:**

We describe the clinical characteristics, pathological features, and choice of examination methods of a patient with TLC in the periorbital region. And we use the radical radiotherapy to treat this case.

**Outcomes:**

There are no progress or metastasis after 2-years follow-up.

**Conclusion:**

Radiotherapy is a good option for patients with TLC if the patient refuses surgery or fails to achieve a satisfactory tumor-free margin or relapses after surgery.

## 1. Introduction

Trichilemmal carcinoma (TLC) is an uncommon malignant adnexal tumor that originates from the external root sheath of hair follicle.^[1]^ The TLC is a rare tumor that usually occurs on sun-exposed skin, which rarely occurs in the periorbital region.^[2]^ It is considered a carcinoma of low malignancy for presenting low recurrence and rare metastasis. And it is considered to be low malignant.^[3]^ The diagnosis is based on histopathological examination on Hematoxylin–Eosin staining and immunohistochemistry.^[4]^ The treatment recommended in the literature is exclusively surgery or micrographic Mohs surgery (MMS) with enough tumor-free margin.^[5,6]^ We report a case of a patient with recurrent TLC of the periorbital region after surgery treated with radiotherapy.

## 2. Case report

**Figure 1.**
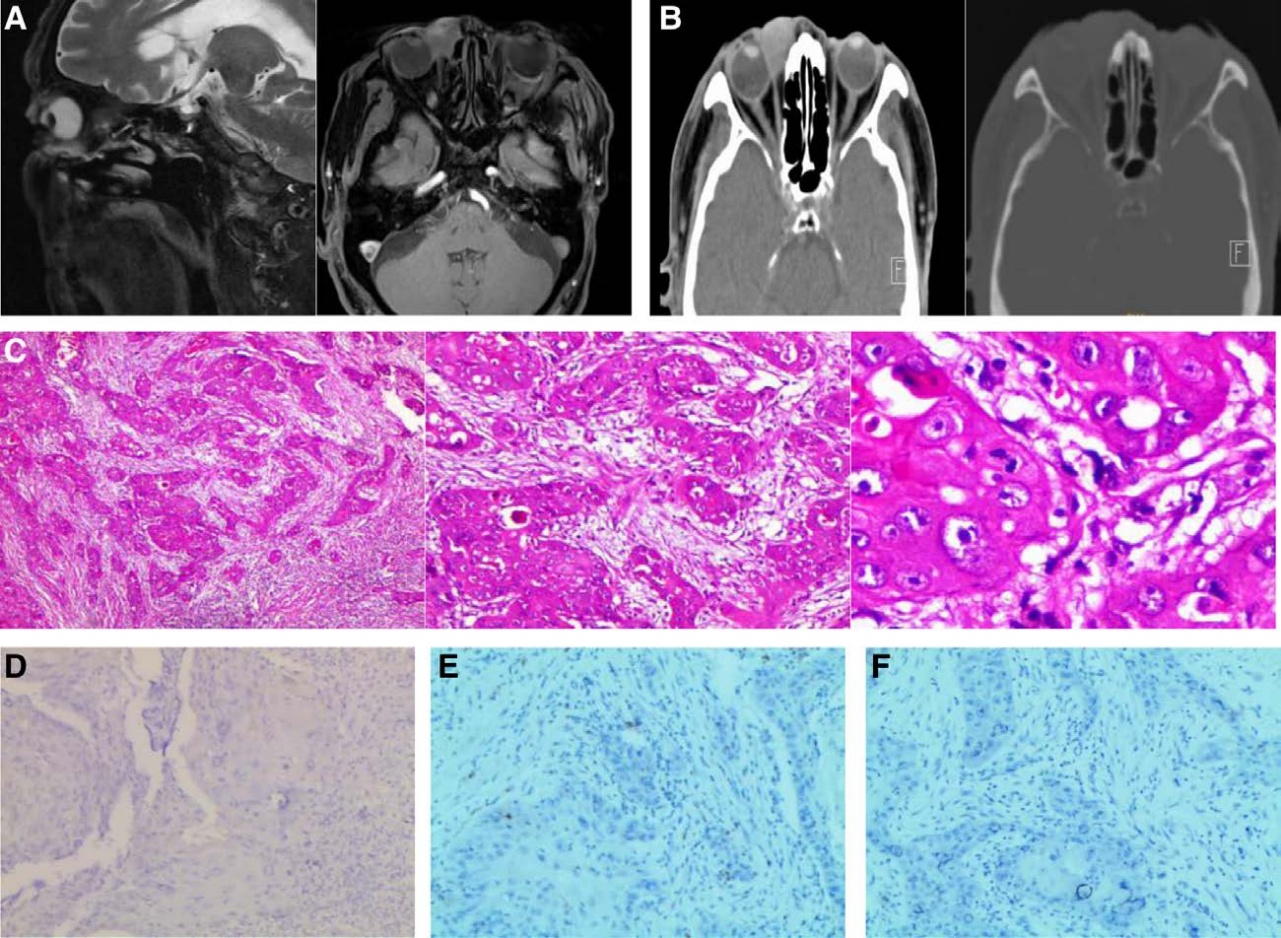
MRI (A): It demonstrated soft tissue thickening and uneven enhancement in the right upper eyelid and lacrimal sac area. CT scan (B): It demonstrated thickening of right eyelid skin, shadow soft tissue mass in the medial part of the right orbit. HE staining (C original magnification × 40, ×100, ×400): It showed obvious tendency of keratosis of hairy root sheath. The tumor cell showed invasive growth, the nucleus is large, the staining is deep, there are pathological mitotic images, the cytoplasm is transparent. The cells around the lobule of the tumor were palisade, and vacuoles were formed in the nucleus. It was negative for EMA, S100 and CEA in immunohistochemical staining (D, E, F respectively). CT = computed tomography, MRI = magnetic resonance imaging.

**Figure 2.**
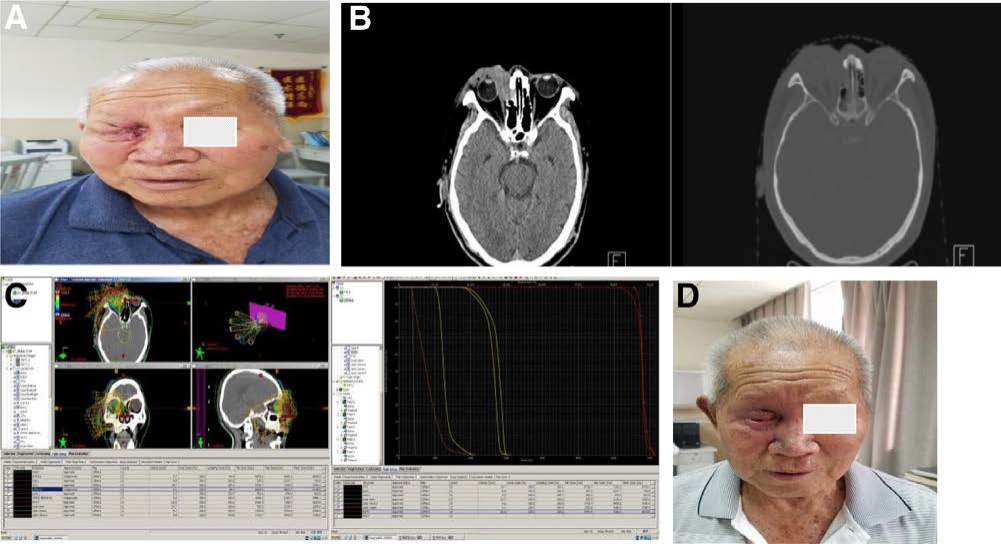
(A) The patient had a recurrence palpable, nontender mass on the right eyelid. (B) CT scan: It demonstrated the soft tissue mass recurred in the medial part of the right orbit, and medial orbital bone destruction could be seen. (C) The Radiation dose curve and DVH diagram. (D) The wound and ulcer of the patient on the right obit were healing after 66 Gy radiotherapy, with mild cutaneous reaction. CT = computed tomography.

**Figure 3.**
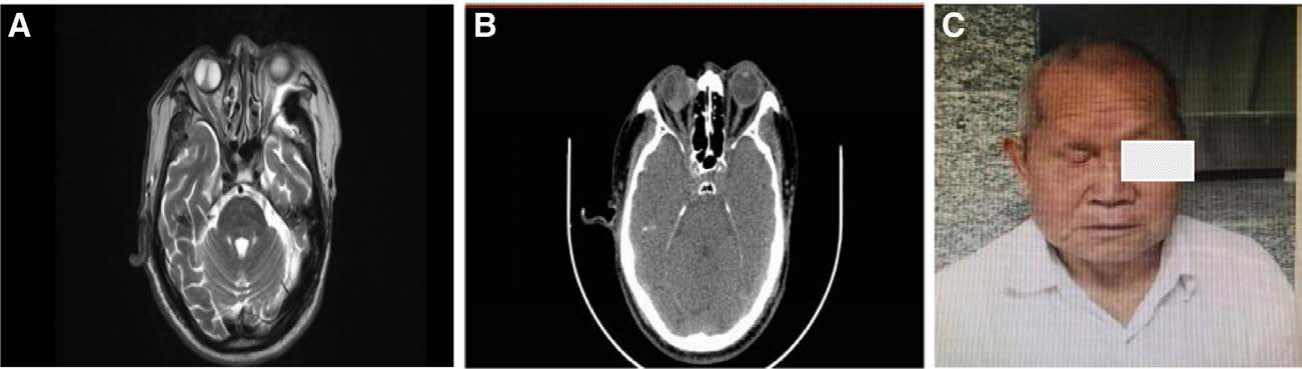
MRI (A): It demonstrated soft tissue thickening but no obvious enhancement in the right upper eyelid and lacrimal sac area. CT scan (B): It demonstrated the thickened soft tissue of the right eyelid was obviously alleviated than that before. (C) The patient after 2 years follow-up. CT = computed tomography, MRI = magnetic resonance imaging.

A 88-year-old man found a palpable, movable nodule on the medial part of the right orbit 3 month ago. He had symptoms of pain and some redness and swelling of right eyelid. His vision was unchanged. He received the treatment of Levofloxacin eye drops. Two months ago he noticed a growing tumor with poor activity, about 2 × 2 cm in size, on the medial part of his right orbit. He accepted computed tomography (CT) scan and magnetic resonance imaging in February 2019. The patient underwent a surgical excision. The excised mass material were sent to the pathology clinic. The diagnosis of trichilemmal carcinoma is established by histopathological examination on Hematoxylin–Eosin staining and immunohistochemical stains. On immunohistochemical staining, the tumor cells were negative for EMA, S100, and CEA (Fig. 1). The resection margins were clear of tumor. But the incision healed poorly after operation. His eyesight was started to decline seriously to a vision of just a sense of light. The patient was referred to us for treatment 4 months after surgery. Swelling of the right eyelid was apparent. Slit-lamp examination revealed a protruding lesion with small ulcer the medial part of his right orbit. Then he was scanned by CT in June 2019 again. The CT scan showed a tumor of the periorbital region, measuring about 2.0 × 1.7 cm. The TLC recurred. The patient refused more surgical excision and chemotherapy due to his age. So we considered to give radical radiotherapy for the recurred TLC instead of a postoperative assistance radiotherapy. Then he received intensity-modulated radiationtherapy (IMRT) radiotherapy with a total dose of 66 Gy. There were no remarkable complications during radiotherapy. The mass was significantly reduced at the end of radiotherapy with mild cutaneous reaction (Fig. 2). Two years later (July 2021), the patient was admitted head, neck, chest, abdomen CT scan, no progress or metastasis was detected, the lesion was obviously reduced but still could be seen on the CT scan and magnetic resonance imaging (Fig. 3).

## 3. Discussion

Malignant hair follicle tumors account for 1% of the adnexal cancer,^[3]^ and trichilemmal carcinoma is rare. It is characterized by asymptomatic exophyte or polypoid mass. It was firstly described as a clinical entity by Headington in 1976.^[7]^ In most patients, the tumor were mostly located in the sunlight exposure areas such as the face, neck, trunk, limbs, and scalp,^[8–10]^ but rarely appeared on the periorbital region.^[2]^

A comprehensive literature review was conducted by searching the PubMed database using the keywords trichilemmal carcinoma, tricholemmal carcinoma, tricholemmocarcinoma, neoplasms and periorbital region. We used The American Joint Committee on Cancer (AJCC) 7th edition, Carcinoma of the Eyelid Staging System 9 to describe the behavior of the tumors and management strategies deployed for TLC case on the periorbital region.^[11]^ We found that most cases on the periorbital region were Stage I or II and most tumors received local surgical excision, some received MMS or methylaminolevulinate-photodynamic therapy.^[5,11–18]^ There are no published guidelines on the follow-up after treatment of patients with TLC.

Trichilemmal carcinoma on the eyelid was first reported in 1993.^[12]^ It typically affects elderly people with a 3:2 male to female ratio, and is rare in young patients.^[2]^ TLC has low grade malignancy and local invasiveness, local recurrence and metastasis are rare.^[3]^ A report goes that TLC occurs on the basis of skin squamous cell carcinoma in the same site.^[13]^ It has also been reported that TLC has been found at the same time in the treatment of breast cancer.^[14]^ Until now the pathogenesis remains unclear, the possible risk factors are ultraviolet radiation, gene disorders, immunosuppression, scars et al In this case, TLC was diagnosed by histopathologic examination of hematoxylin-eosin stained slides,^[5,15]^ and negative EMA, S-100, CEA on immunohistochemistry ruled out squamous cell carcinoma, basal-cell carcinoma, melanoma.^[13]^

The recommended treatment for TLC is local surgical excision or MMS with enough tumor-free margins. Recurrence or metastasis of this neoplasm was seldom reported in the medical literature.^[5]^ Periodic surveillance is generally sufficient after free margins. There is no suggestion of adjuvant therapy.^[16]^

Radiotherapy has been used both as a primary treatment and adjuvant treatment. A case of adjuvant radiotherapy used on a recurrence of TLC lesion after resection of basal cell carcinoma in left clavicular region. He received a dose of 4320 cGy radiotherapy, but recurred 2 years later.^[17]^ Local orbital and periorbital invasion was described in another case. The tumor recurred and invaded the orbit after a dose of 60 Gy irradiation. Then managed local surgical excision with 5 mm excision margin. No relapse was detected during a 29-month follow-up of the patient.^[18]^

We report here a case of recurrent TLC involving local orbital invasion. It is speculated that the ideal 5 mm safe surgical margin may not be achieved at the first operation due to the limitation of the anatomical site. Four months later, he invaded the intraorbital structures and it was difficult to ensure sufficient 5 mm surgical margin if reoperation, and the patient refused reoperation and chemotherapy due to advanced age. In this case we gave IMRT radiotherapy. Considering that the 60 Gy dose cannot reach the radical dose, we gave a higher dose of 66 Gy (conventional segmentation, 33 times) of 6 MVX photon irradiation (Varian) to improve the local control rate. Since this patient had been complicated with unhealed skin ulcer which suggested that the skin was invaded, 0.5 cm compensation membrane was added to the surface to increase the dose of skin surface during radiotherapy. Because the tumor was too close to the right eye lens, and he had been almost blindness, fully inform the risk of blindness before radiotherapy and sign the consent form. We outlined GTV (tumor target area), with 3 mm external expansion to form PTV (planned target area). The average dose of 95% prescription dose should be wrapped around 100% PTV. At the end of radiotherapy, the ulcer healed and the tumor was significantly reduced. There was no progress or metastasis after 2 year follow-up.

Other treatment include cisplatin and cyclophosphamide chemotherapy and other drug treatments (5% imiquimod).^[19–21]^ Nevertheless, these treatment continue to be utilized as an adjunct treatment for surgery or metastatic disease.^[2]^

TLC is rare on the periorbital region. Complete tumor-free excision margin is important for reducing local recurrence and metastasis. The present report shows a recurrent TLC case treated with radiotherapy (Table 1). Although surgical treatment is recommended but radiotherapy is not clearly recommended in the treatment, adequate IMRT is still a good option if the patient refuses surgery or fails to achieve a satisfactory tumor-free margin or relapses after surgery. Although long-term follow-up about relapse and metastasis need more research and data.

**Table 1:** Comparison of the present case with previously published case reports.

Authors	Location	Patient age	Patient gender	Publication country	Medical history	Diagnostic test	Treatments	Outcomes	Follow up
Dailey JR, 1993	Left upper eyelid	95	Woman	American	Breast cancer	Histopathological examination	Excision	No recurrence	No
Miguel Roismann, 2011	Anterior thoracic wall	35	Man	NK	Basal-cell carcinoma	Histopathological examination	Excision then recurrence then chemotheray and 4320 cGy radiotherapy	Recurrence and metastasis	More than 6 yr
Tomifuji M, 2004	Left upper eyelid	65	Man	Japan	No	Histopathological examination	Mohs micrographic surgery.	No recurrence	29-mo
This case	Left periorbital region	88	Man	China	No	Histopathological examination	Excision then recurrence then 6600cGy radiotherapy	No recurrence	2 yr

## Author contributions

**Data curation:** Qin Xu, Lang He.

**Investigation:** Qin Xu.

**Resources:** Shan Gao.

**Supervision:** Shan Gao, Yang Lan, Lang He.

**Writing – original draft:** Qin Xu.

**Writing – review & editing:** Shan Gao, Yang Lan, Lang He.
